# Supplementary material for: MetaRibo-Seq measures translation in microbiomes
Source: Nat Commun. 2020 Jun 29;11:3268. doi: 10.1038/s41467-020-17081-z (PMC7324362; doi:10.1038/s41467-020-17081-z)
Supplement: Supplementary file 10 — Supplementary Data 7 [file 41467_2020_17081_MOESM10_ESM.zip › File2/Confidence_High_Taxonomy/320859_out.krona.html]

Javascript must be enabled to view this page.

members
magnitude
magnitudeUnassigned
count
unassigned
taxon
rank

320859\_out

7

2
7
superkingdom

phylum
7
976

class
7
200643

order
171549
7

171552
7
family

7
577309
genus

1263095
1

SRS049712\_contig\_number\_12653
species

species

SRS013687\_contig\_number\_20317SRS016541\_contig\_number\_contig-100\_6746.80283SRS023914\_contig\_number\_22328SRS1041138\_contig\_number\_8935SRS105082\_contig\_number\_11776SRS143876\_contig\_number\_14050
454154
6
